# A Rare Case of Diffuse Idiopathic Pulmonary Neuroendocrine Cell Hyperplasia: A Case Report

**DOI:** 10.7759/cureus.62527

**Published:** 2024-06-17

**Authors:** Sindhu C Pokhriyal, Nisha Sapkota, Muthanna Mohammed Hasan Al-Ghuraibawi, Muhammad N Pasha, Ahmad Ali Khan, Hadeeqa Idris, Kalpana Panigrahi

**Affiliations:** 1 Internal Medicine, One Brooklyn Health-Interfaith Medical Center, New York, USA; 2 Medicine, One Brooklyn Health-Interfaith Medical Center, New York, USA; 3 Pulmonary and Critical Care Medicine, One Brooklyn Health, New York, USA; 4 Internal Medicine, Shifa International Hospital, Islamabad, PAK

**Keywords:** nodule, pulmonary, lung, neuroendocrine, dipnech

## Abstract

Diffuse idiopathic pulmonary neuroendocrine cell hyperplasia (DIPNECH) is a rare pulmonary disease characterized by the diffuse proliferation of neuroendocrine cells in the bronchial epithelium. It is considered a preinvasive precursor to carcinoid tumors and usually presents with obstructive symptoms. We present the case of a 71-year-old female, non-smoker, with a past medical history of asthma, osteoarthritis, allergic rhinitis, and hyperlipidemia who was referred to the pulmonology clinic in view of incidental chest CT findings of multiple pulmonary nodules. Physical examination and labs were unremarkable. CT of the chest showed scattered multiple noncalcified pulmonary nodules with a 10 mm dominant nodule in the inferior right middle lobe and several subcentimeter hypodensities in the left and right lobes of the lung. A PET scan confirmed the CT findings along with no abnormal hypermetabolic activity to suggest malignancy. The patient was followed up in the pulmonology clinic at six months, 12 months, and then 18 months. At 18 months owing to a slight increase in the size of the largest lung nodule, a CT-guided biopsy done was conclusive of a carcinoid. The tumor cells were positive for synaptophysin, chromogranin, insulinoma-associated protein 1 (INSM-1), and thyroid transcription factor 1 (TTF-1). The Ki-67 (Keil) index was <1%. A video-assisted thoracic surgery with right middle lobectomy along with mediastinal lymph node dissection was then done, and the patient was found to have stage pT1aN0 typical carcinoid tumor (1.0 cm), with multiple carcinoid tumors and neuroendocrine hyperplasia, consistent with DIPNECH. She has been under clinical follow-up for over three years at present and continues to be asymptomatic with complete remission following surgery. DIPNECH primarily affects middle-aged, non-smoking females who present with cough and dyspnea, and diagnosis is often delayed due to clinical features overlapping with those of obstructive lung disease. Imaging shows lung nodules, ground-glass opacities, and/or mosaic attenuation. Due to the rarity of the conditions, there are no established clinical trials, and therefore, there is a need to establish guidelines.

## Introduction

Neuroendocrine cells constitute a part of the pulmonary epithelium. Hyperplasia of neuroendocrine cells can be either reactive or idiopathic [1]. Reactive neuroendocrine cell hyperplasia is observed in diverse conditions associated with hypoxia [2]. The idiopathic form is specifically referred to as diffuse idiopathic pulmonary neuroendocrine cell hyperplasia (DIPNECH) [3].

The World Health Organization (WHO) defines it as a generalized proliferation of scattered single cells, small nodules (neuroendocrine bodies), or linear proliferations of pulmonary neuroendocrine cells that may be confined to the bronchial and bronchiolar epithelium [4]. DIPNECH is an uncommon pulmonary condition marked by the widespread growth of neuroendocrine cells in the bronchial and bronchiolar epithelium, without penetration through the basement membrane. When there is a breach in the basement membrane, the lesion is termed a carcinoid tumorlet if the nodule diameter is ≤5 mm and a carcinoid tumor if the nodule diameter exceeds 5 mm [5] [6]. However, its recognition is on the rise, attributed to the current uptick in lung cancer screening efforts. DIPNECH primarily affects women, with a female-to-male ratio of about 10:1, and typically manifests around the age of 58 years [7].

The management of DIPNECH is not a very commonly discussed topic. The current case describes a typical presentation of a patient with DIPNECH and its management and outcome.

## Case presentation

We present the case of a 71-year-old woman with a medical history of asthma, osteoarthritis, allergic rhinitis, and hyperlipidemia, who was a non-smoker. She had a past surgical history of bilateral total knee replacement. She initially sought medical attention due to persistent dysphagia for a year and was subsequently referred to both otolaryngology and gastrointestinal specialists. She exhibited no other respiratory symptoms, orthopnea, paroxysmal nocturnal dyspnea, gastroesophageal reflux symptoms, nasal complaints, or constitutional symptoms. Following a CT scan of the chest and abdomen, incidental findings of multiple pulmonary nodules prompted her referral to the pulmonology clinic. Of note, her family history included a sister diagnosed with breast cancer and a deceased brother who passed away due to throat cancer, both before 50 years of age.

On physical examination, the patient was found to be afebrile and hemodynamically stable. There were no signs of respiratory distress, and her oxygen saturation while breathing room air was 98%. Additionally, both cardiac and pulmonary auscultation revealed normal findings. Laboratory findings were unremarkable as presented in Table 1. 

**Table 1:** Patient's laboratory findings on admission

Laboratory test	Normal range	Results
White blood cell	4.5-11.0×10^3^/uL	3.8
Hemoglobin	11.0-15.0 g/dL	10.7
Hematocrit	35-46%	33.4
Mean corpuscular volume	80-100 fL	80.5
Platelets	130-400×10^3^/uL	171
Blood urea nitrogen	9.8-20.1 mg/dL	13
Creatinine	0.57-1.11 mg/dL	0.6
Estimated glomerular filtration rate	≥90 mL/min/1.73 m^2^	>90
Potassium	3.5-5.1 mmol/L	3.7
Sodium	133-145 meQ/L	140
Phosphorus	2.3-4.7 mg/dL	3.4
Magnesium	1.6-2.6 mg/dL	2.1
Calcium	8.4-10.5 mg/dL	9.1
Vitamin D	30-100 ng/mL	22.8
Prothrombin time	9.8-13.4 seconds	10.3
International normalized ratio	0.85-1.15 ratio	0.92
Troponin I	0-17 ng/L	0
Partial thromboplastin time	24.9-35.9 seconds	31.7
Brain natriuretic peptide	10-100 pg/mL	11.3

**Figure 1:**
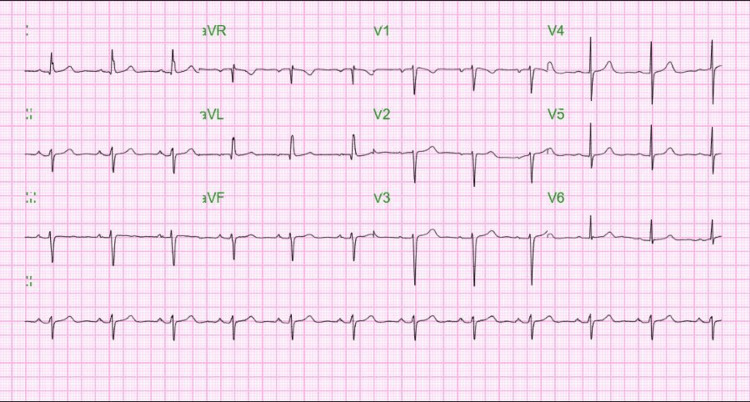
Electrocardiogram findings showing left-axis deviation and left anterior fascicular block

An electrocardiogram (EKG) showed a ventricular rate of 75 beats per minute and left-axis deviation with left anterior fascicular block as shown in Figure 1.

**Figure 2:**
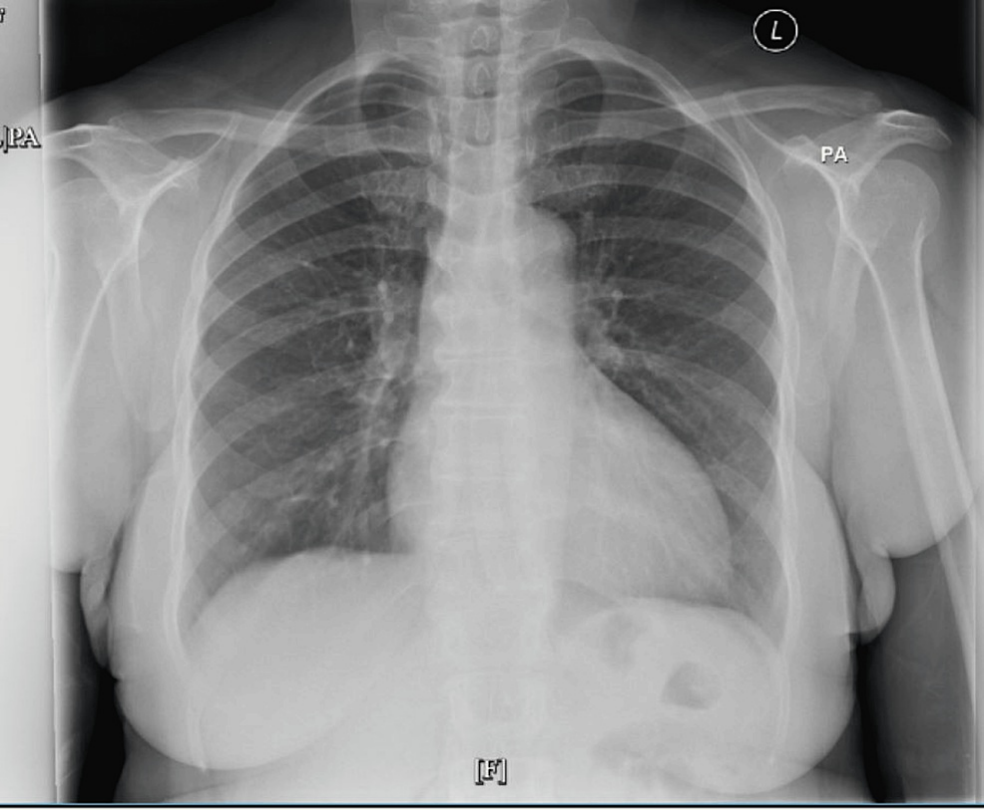
Chest X-ray showing normal findings

**Figure 3:**
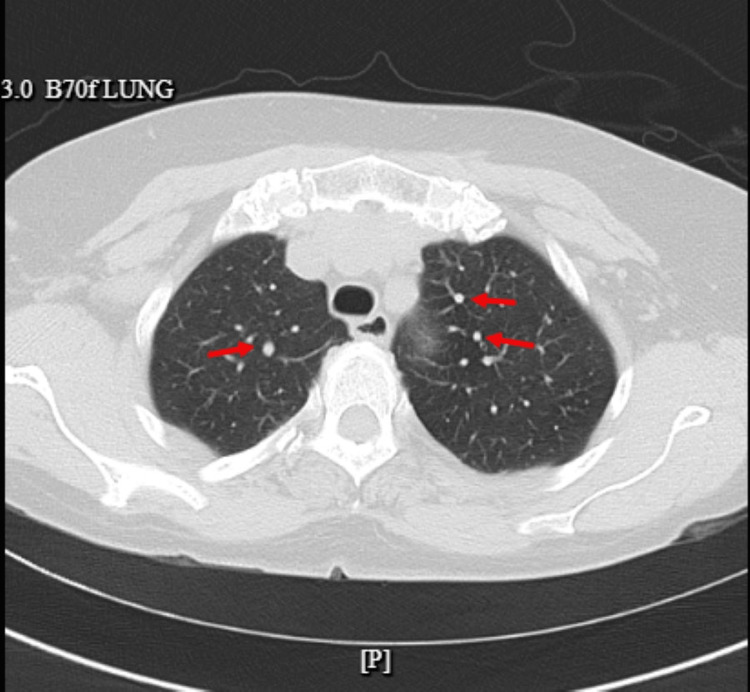
CT of the chest showing multiple pulmonary nodules as pointed out by the red arrows

**Figure 4:**
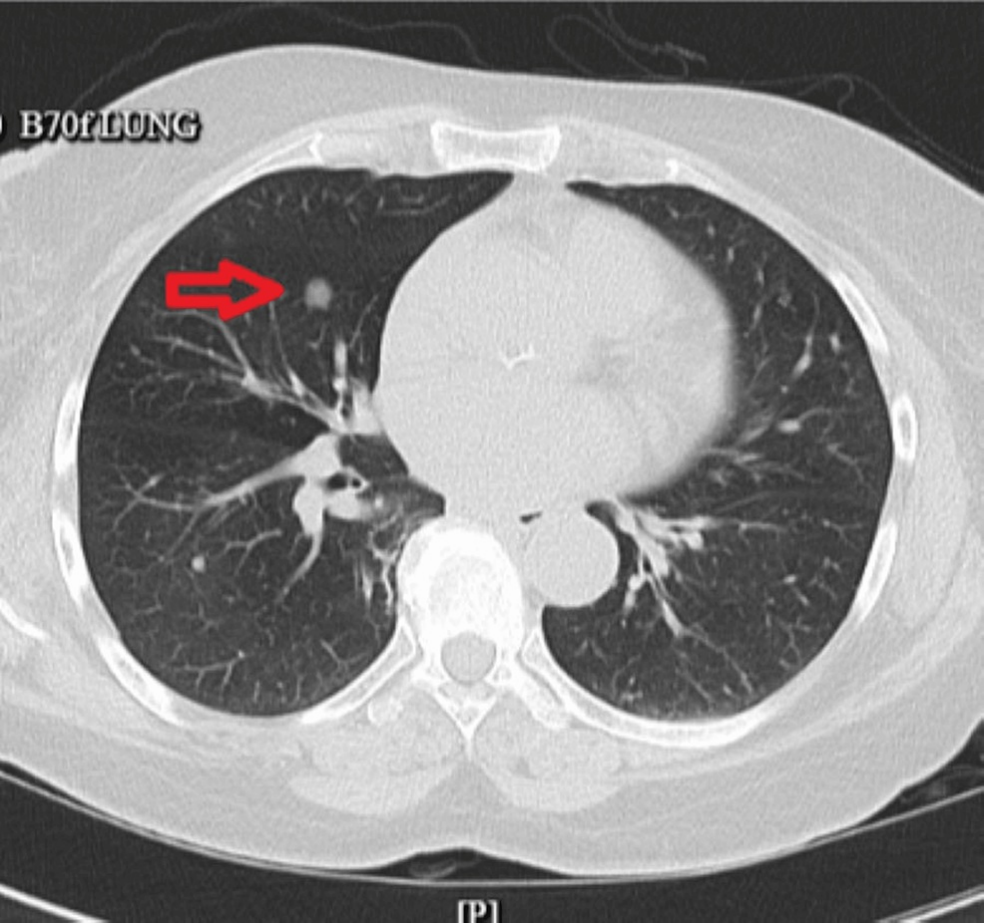
CT of the chest showing a dominant 10 mm pulmonary nodule

Chest X-ray was reported normal, and CT of the chest showed scattered multiple noncalcified pulmonary nodules with the most prominent nodule measuring 10 mm in the inferior right middle lobe and several subcentimeter hypodensities in the left and right lobes of the lung (as shown in Figure 2, Figure 3, and Figure 4, respectively).

**Figure 5:**
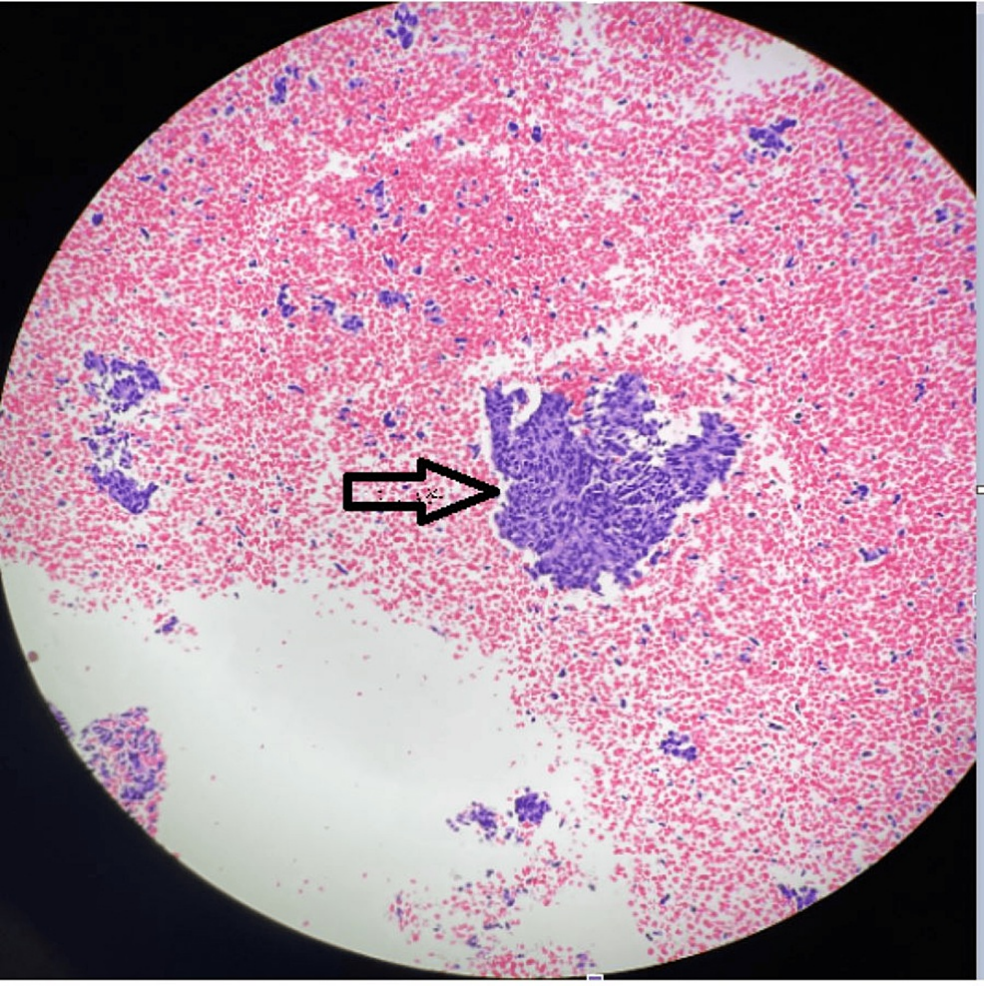
Lung node biopsy with the arrow pointing at a carcinoid tumorlet

**Figure 6:**
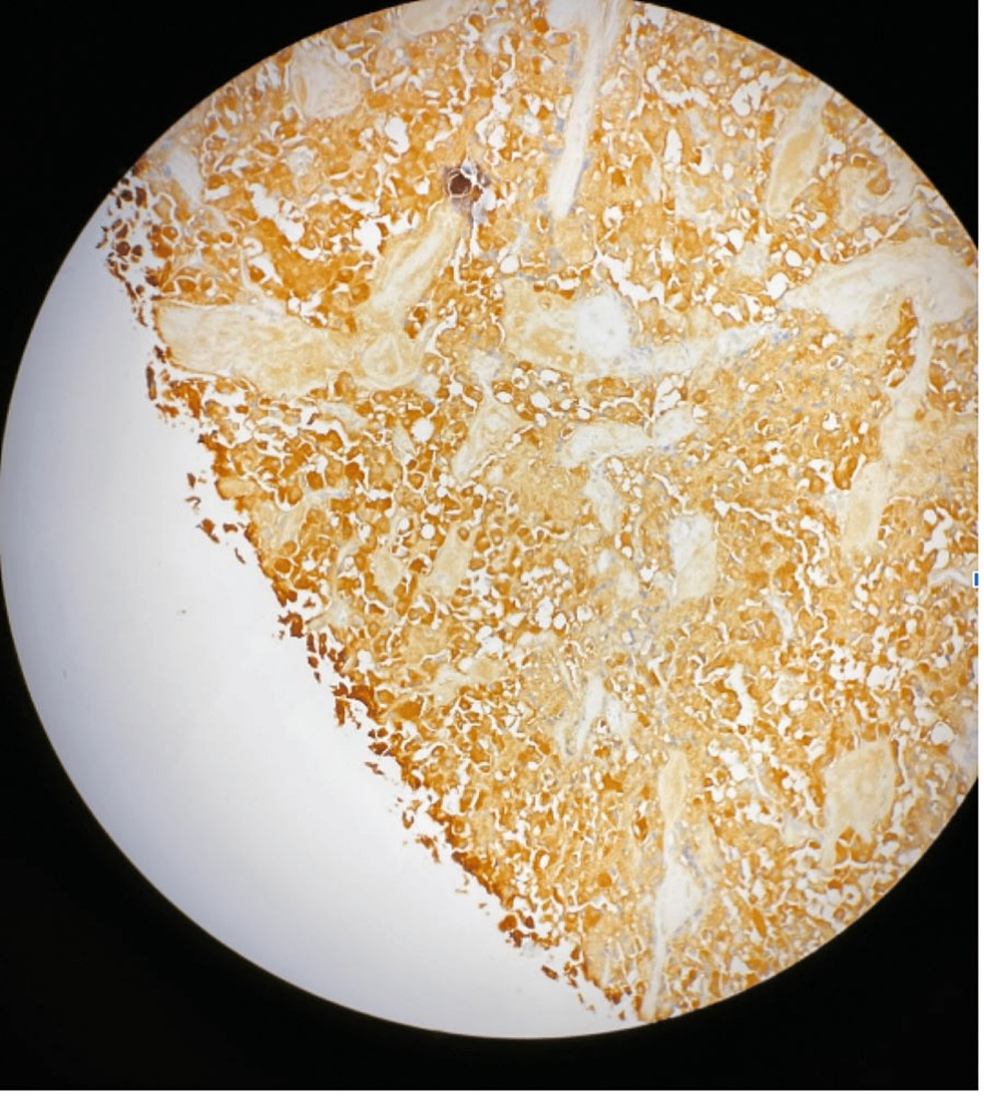
Tumor cells showing strong synaptophysin expression

At this time, the patient was already on a combination of long-acting beta-2 agonist and inhaled corticosteroids for the management of her moderate persistent asthma. She was followed up in the pulmonary clinic at six months, 12 months, and then 18 months. At 18 months owing to a slight increase in the size of the largest lung nodule, a CT-guided biopsy showed findings consistent with DIPNECH (Figure 5 and Figure 6). The tumor cells were positive for synaptophysin, chromogranin, insulinoma-associated protein 1 (INSM-1), and thyroid transcription factor 1 (TTF-1). The Ki-67 (Keil) index was <1% (Figure 6).

A video-assisted thoracic surgery with right middle lobectomy along with mediastinal lymph node dissection was then done, and the patient was found to have stage pT1aN0 typical carcinoid tumor (1.0 cm), with multiple carcinoid tumor and neuroendocrine hyperplasia, consistent with DIPNECH. She has been under clinical follow-up for over three years at present and continues to be asymptomatic with complete remission following surgery.

## Discussion

The exact incidence is unknown due to the rarity of DIPNECH, but it is being recognized with increasing frequency [8]. About 300 cases have been reported in the literature [9]. Among patients with well-differentiated lung neuroendocrine tumors, the prevalence of DIPNECH may be as high as 20% [5]. It primarily affects middle-aged females, with the average age of diagnosis being 63 years [8-10]. Of note also is the fact that a majority of the patients affected with DIPNECH are non-smokers [6,9,10]. In our scenario, the patient is elderly and does not have a history of smoking. In some cases, a familial link has been observed with two sisters affected by DIPNECH [11]. However, there is a lack of clear evidence supporting family aggregation or germline genetic alterations in this particular condition [11,12]. In our case, the patient's sister has experienced breast cancer, and their brother has been diagnosed with throat cancer, without any history of DIPNECH.

The most common symptoms are chronic cough (71%) and dyspnea (44%) [9]. Other symptoms include wheezing, hemoptysis, and home oxygen requirement [6]. Up to 54% may be asymptomatic at presentation, and DIPNECH might be discovered incidentally through chest CT scans or in surgical specimens [7,8]. While most patients previously documented with DIPNECH tend to have a relatively stable clinical course, it is important to note that severe clinical decline can occur in some patients. Therefore, it is advisable to achieve a prompt diagnosis and initiate treatment for optimal outcomes. In our case, the patient solely presented with complaints of dysphagia, and the CT findings revealed the presence of DIPNECH [7,8].

Radiologically, DIPNECH lesions almost always exhibit bilateral lung nodules in 93% of cases, displaying characteristic signs of airway disease like bronchial wall thickening and bronchiectasis, leading to apparent air trapping [9,13,14]. Our patient also presented with multiple bilateral pulmonary nodules. The pulmonary function of individuals with DIPNECH is commonly identified by obstructive or mixed obstructive/restrictive defects, and instances of solely restrictive patterns are infrequent [6,10,13]. Despite the gradual and indolent progression of the disease, utilizing spirometry alongside high-resolution computed tomography (HRCT) stands as a promising means to closely track patients for any indications of advancement [13]. Our patient had a past medical history of asthma and had an obstructive pattern on the pulmonary function test.

Histologically, DIPNECH is characterized in diverse forms upon biopsy. It is recognized as a generalized proliferation of scattered neuroendocrine cells, small nodules (neuroendocrine bodies), or a linear proliferation of pulmonary neuroendocrine cells. The presence of more prominent nodules may signify proliferations extending beyond the basement membrane, resulting in the formation of tumorlets (distinct aggregates of neuroendocrine cells <5mm in diameter) or larger carcinoid tumors (nodules >5mm in diameter) [7,13]. While surgical lung stands as the diagnostic gold standard, transbronchial lung biopsy may provide a confident diagnosis of DIPNECH syndrome in a suitable clinical and radiological context [7]. 

Establishing a diagnosis of DIPNECH necessitates a thorough integration of clinical, functional, and imaging data, in conjunction with the histological confirmation of constrictive bronchiolitis that mirrors neuroendocrine cell proliferation [7].

When the symptoms require pharmacological intervention, the initial approach involves the initiation of inhaled beta-2 agonists (80%), inhaled corticosteroids (49%), antitussive agents, and systemic steroids(49%) [9]. In some studies, systemic corticosteroids are discouraged due to their ineffectiveness and significant toxicity [15]. If symptoms persist despite these interventions, the possibility of conducting a trial with somatostatin analogs (SSAs) may be considered. This has demonstrated the ability to ameliorate chronic respiratory symptoms and enhance pulmonary function test results [16,17]. Our patient was on long-acting beta-2 agonists and inhaled corticosteroids for asthma, and her symptoms stayed stable after the video-assisted thoracoscopic surgery (VATS) procedure.

The mammalian target of rapamycin (mTOR), an intracellular protein kinase within the phosphoinositide-3 kinase family, is a pivotal component in the cell signalling pathway that contributes to the development of neuroendocrine tumors. Everolimus, an mTOR inhibitor, by modulating this pathway, has been demonstrated to enhance progression-free survival and exhibit an acceptable tolerability profile [18,19]. Nonetheless, considering their notable toxicity, including pulmonary effects, some studies suggest that initiating mTOR inhibitors solely for relieving respiratory symptoms may not be advisable. Instead, it is preferable to reserve their use for DIPNECH patients facing progressive respiratory failure [15].

For patients with localized neuroendocrine tumors, surgical resection is the preferred treatment approach, assuming adequate pulmonary reserve. For patients whose condition does not permit complete resection and for exceptional low-grade cases where the lesion is entirely endobronchial, transbronchoscopic resection may be an alternative [20]. The role of adjuvant therapy following the complete resection of a lung neuroendocrine tumor is unclear, primarily attributed to the absence of prospective randomized trials [20].

## Conclusions

DIPNECH is an under-recognized pulmonary disease that can significantly impact the quality of life in a lot of patients and frequently is misdiagnosed as chronic obstructive pulmonary disease (COPD). More research is needed to establish evidence-based guidelines for diagnosis and management. A national patient registry would help advance our understanding of this rare entity.

## References

[1] Noguchi M, Furukawa KT, Morimoto M (2020). Pulmonary neuroendocrine cells: physiology, tissue homeostasis and disease. Dis Model Mech.

[2] Garg A, Sui P, Verheyden JM, Young LR, Sun X (2019). Consider the lung as a sensory organ: a tip from pulmonary neuroendocrine cells. Curr Top Dev Biol.

[3] Koliakos E, Thomopoulos T, Abbassi Z, Duc C, Christodoulou M (2017). Diffuse idiopathic pulmonary neuroendocrine cell hyperplasia: a case report and review of the literature. Am J Case Rep.

[4] Travis WD, Brambilla E, Burke AP, Marx A, Nicholson AG (2015). Introduction to the 2015 World Health Organization classification of tumors of the lung, pleura, thymus, and heart. J Thorac Oncol.

[5] Alves AP, Barroso A, Dias M (2020). Diffuse idiopathic pulmonary neuroendocrine cell hyperplasia: a clinical case. Eur J Case Rep Intern Med.

[6] Sun TY, Hwang G, Pancirer D (2022). Diffuse idiopathic pulmonary neuroendocrine cell hyperplasia: clinical characteristics and progression to carcinoid tumour. Eur Respir J.

[7] Rossi G, Cavazza A, Spagnolo P (2016). Diffuse idiopathic pulmonary neuroendocrine cell hyperplasia syndrome. Eur Respir J.

[8] Myint ZW, McCormick J, Chauhan A, Behrens E, Anthony LB (2018). Management of diffuse idiopathic pulmonary neuroendocrine cell hyperplasia: review and a single center experience. Lung.

[9] Almquist D, Cabrera A, Sonbol MB (2019). DIPNECH: the Mayo experience. J Clin Oncol.

[10] Nassar AA, Jaroszewski DE, Helmers RA, Colby TV, Patel BM, Mookadam F (2011). Diffuse idiopathic pulmonary neuroendocrine cell hyperplasia: a systematic overview. Am J Respir Crit Care Med.

[11] Cabezón-Gutiérrez L, Khosravi-Shahi P, Palka-Kotlowska M, Custodio-Cabello S, García-Martos M (2019). Diffuse idiopathic pulmonary neuroendocrine cell hyperplasia: review of the literature and a single-center experience. Cureus.

[12] Gorospe L, Muñoz-Molina GM, Farfán-Leal FE, Cabañero-Sánchez A, García-Gómez-Muriel I, Benito-Berlinches A (2017). Association of diffuse idiopathic pulmonary neuroendocrine cell hyperplasia (DIPNECH) with lung adenocarcinoma: a radiologist's perspective. Lung Cancer.

[13] Shah HV, Shah M, Mahathevan K (2022). Pulmonary function tests as a biomarker in diffuse idiopathic pulmonary neuroendocrine cell hyperplasia patients treated with somatostatin analogues. Cureus.

[14] Chassagnon G, Favelle O, Marchand-Adam S, De Muret A, Revel MP (2015). DIPNECH: when to suggest this diagnosis on CT. Clin Radiol.

[15] Samhouri BF, Halfdanarson TR, Koo CW, McCarthy C, Yi ES, Thomas CF, Ryu JH (2023). DIPNECH: pragmatic approach, uncertainties, notable associations, and a proposal for an improved definition. Endocr Relat Cancer.

[16] Al-Toubah T, Strosberg J, Halfdanarson TR (2020). Somatostatin analogs improve respiratory symptoms in patients with diffuse idiopathic neuroendocrine cell hyperplasia. Chest.

[17] Chauhan A, Ramirez RA (2015). Diffuse idiopathic pulmonary neuroendocrine cell hyperplasia (DIPNECH) and the role of somatostatin analogs: a case series. Lung.

[18] Simon N, Negmeldin M (2022). Diffuse idiopathic pulmonary neuroendocrine cell hyperplasia presenting as a solitary lung nodule: a rare histopathological diagnosis. Oxf Med Case Reports.

[19] Yao JC, Fazio N, Singh S (2016). Everolimus for the treatment of advanced, non-functional neuroendocrine tumours of the lung or gastrointestinal tract (RADIANT-4): a randomised, placebo-controlled, phase 3 study. Lancet.

[20] Ramirez RA, Thomas K, Jacob A, Lin K, Bren-Mattison Y, Chauhan A (2021). Adjuvant therapy for lung neuroendocrine neoplasms. World J Clin Oncol.

